# Functional trade-offs in cribellate silk mediated by spinning behavior

**DOI:** 10.1038/s41598-019-45552-x

**Published:** 2019-06-24

**Authors:** Peter Michalik, Dakota Piorkowski, Todd A. Blackledge, Martín J. Ramírez

**Affiliations:** 1 Zoological Institute and Museum, University of Greifswald, Loitzer Straße 26, D-17489 Greifswald, Germany; 2 Department of Life Sciences, Tunghai University, Taichung, Taiwan; 3 Department of Biology and Integrated Bioscience Program, The University of Akron, Akron, OH USA; 4 Division of Arachnology, Museo Argentino de Ciencias Naturales - CONICET, Buenos Aires, Argentina

**Keywords:** Evolution, Zoology

## Abstract

Web-building spiders are an extremely diverse predatory group due to their use of physiologically differentiated silk types in webs. Major shifts in silk functional properties are classically attributed to innovations in silk genes and protein expression. Here, we disentangle the effects of spinning behavior on silk performance of the earliest types of capture threads in spider webs for the first time. *Progradungula otwayensis* produces two variations of cribellate silk in webs: ladder lines are stereotypically combed with the calamistrum while supporting rail lines contain silk that is naturally uncombed, spun without the intervention of the legs. Combed cribellate silk is highly extensible and adhesive suggesting that the reserve warp and cribellate fibrils brings them into tension only near or after the underlying axial fibers are broken. In contrast, these three fiber components are largely aligned in the uncombed threads and deform as a single composite unit that is 5–10x stronger, but significantly less adhesive, allowing them to act as structural elements in the web. Our study reveals that cribellate silk can occupy a surprisingly diverse performance space, accessible through simple changes in spider behavior, which may have facilitated the impressive diversification of web architectures utilizing this ancient silk.

## Introduction

Spiders are successful and diverse predators in terrestrial ecosystems^1^ due in part to the production of silk and spinning of aerial webs^2^. Moreover, spider silk is an outstanding biomaterial that has received significant attention^3^. Most prey capture webs consist of non-sticky ampullate silk structural elements and sticky capture threads that adhere to prey. Glue droplet coated viscid silk is most familiar and is the dominant capture silk in most orb- and cob-webs^4^. However, viscid silk evolved recently and many spiders instead use cribellate silk to make webs adhesive. Cribellate silk uses a mesh of very thin, dry nano-fibers around its axial threads to generate adhesion^5,6^. Cribellate silk is laboriously spun from the functionally co-dependent cribellum (a highly transformed pair of spinnerets^7^ with up to 40,000 tiny spigots) and calamistrum (a specialized comb of setae on the hind legs used to pull silk from the cribellum)^5,8,9^. Cribellate threads are composites of several interwoven fiber types. The most prominent fibers are the (1) axial fibers produced by pseudoflagelliform glands, (2) reserve warps (undulating fibers; except Uloboridae^5,10^) and (3) the characteristic mass of nanofibers originating from the cribellum^11^. The reserve warps usually coil into a unique spiral morphology that is thought to enhance extensibility^6,11^. In a classic cribellate thread the nanofibers are combed into characteristic puffs using the calamistrum^5,12^. Removing the calamistrum does not hinder uloborid spiders from using their hind legs to manipulate and produce functional threads, but greatly reduces cribellar puffs, indicating that combing is a crucial stereotyped behavior in cribellate spiders^13^.

Cribellate threads adhere through physical interlock, capillary forces^14^, and van der Waal’s forces so that adhesion depends in part on the total number of cribellate fibrils per length of thread. The puffs produced by combing may increase the total surface area of cribellate fibrils thereby improving adhesion^15,16^. Their composite nature also makes the tensile behaviors of cribellate threads quite complex as the cribellate fibrils are typically very loosely laid down on the axial fibers so that they stretch and deform somewhat independently of the axial fibers themselves^6^, in contrast to viscid silk where the deformation of the glue droplets and the underlying axial fibers is tightly linked^17^. Both the puffs and how the cribellate fibrils are laid down are under behavioral control by spiders. However, the complex cribellate threads are not easily disentangled making it difficult to directly test the effects of each fiber component on thread performance.

Here, we disentangle the effects of spinning behavior on silk performance of the earliest type of capture threads in spider webs. We describe naturally uncombed cribellate threads, produced without intervention of the hind legs, which are incorporated as a structural element within the web of the Otway odd-clawed spider *Progradungula otwayensis*. We show how the different spinning behaviors allow the same silk to be used both as a strong, stiff structural element and as a highly extensible and adhesive capture thread. Our data suggest that the uncombed thread represents a new functional type of silk thread due to the clear differences in mechanical and adhesive properties compared to combed cribellate threads.

## Results

**Figure 1:**
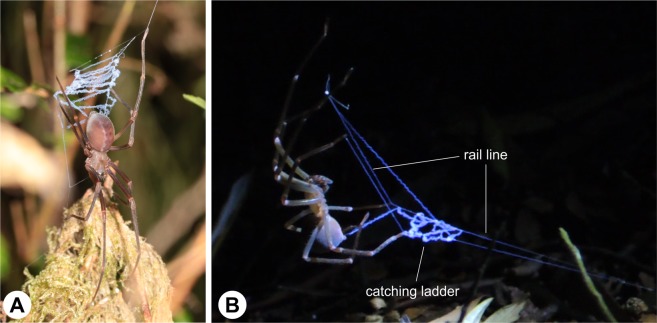
(**A**) Individual of *P*. *otwayensis* in hunting position holding the sticky cribellate catching ladder. (**B**) Individual of *P*. *otwayensis* combing the sticky cribellate ladder thread. The cribellate character of the rail lines is clearly visible by its blue appearance.

**Figure 2:**
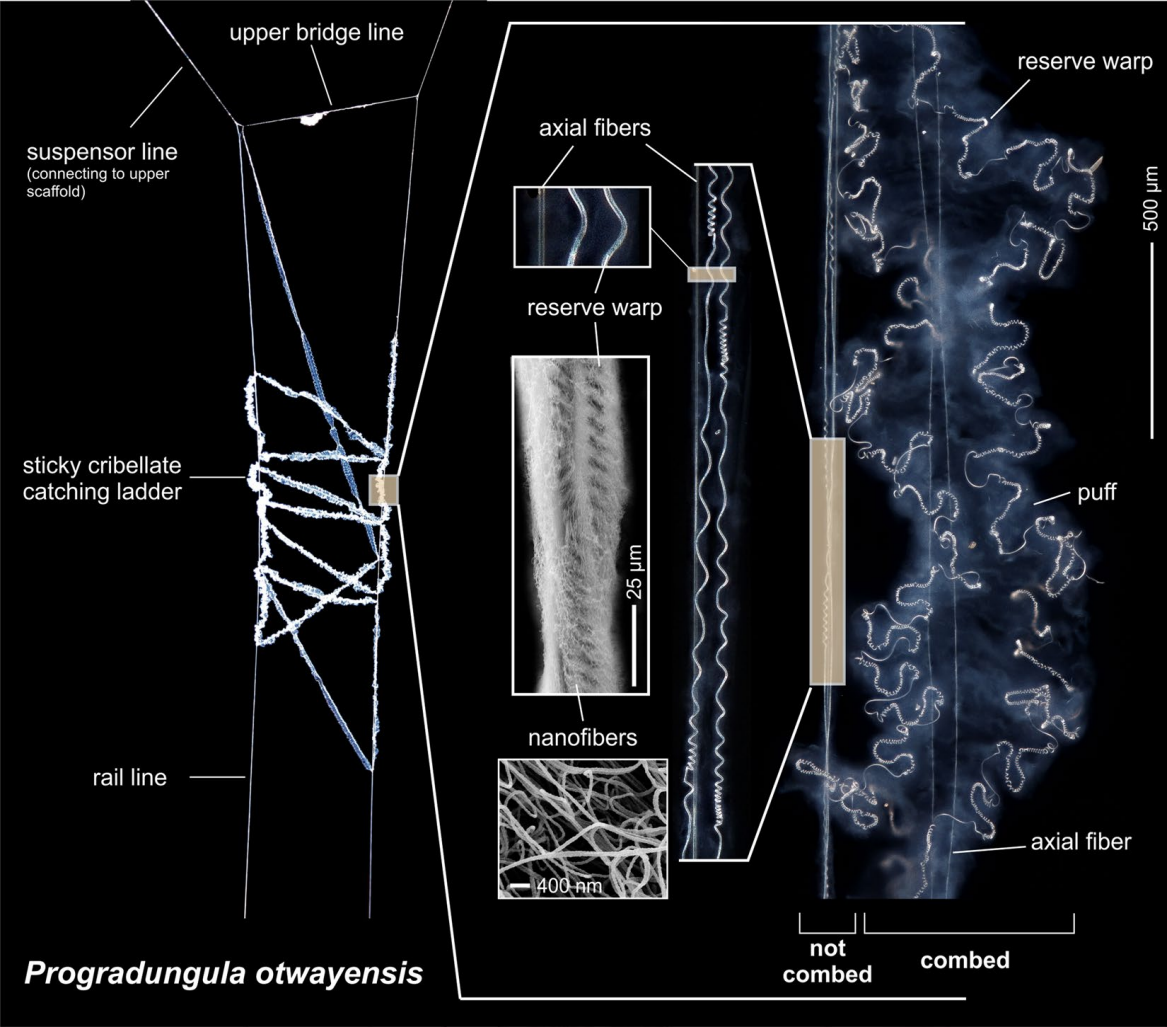
The sticky cribellate catching ladder in the web of *P*. *otwayensis* is attached to lateral rail lines representing uncombed cribellate silk, which consist of two axial fibers and reserve warps embedded in nanofibers (SEM images). The right part of the figure illustrates the size difference of combed and uncombed silk.

*P*. *otwayensis* webs consist of a signal line, upper scaffolding and a prominent catching ladder^18^ (Fig. 1A). The catching ladder consists of combed cribellate silk, which appears as a very wide band (>500 µm) with highly coiled reserve warps and ‘puffs’ of nanofibers (Fig. 2). This sticky ladder is mounted between two rail lines that connect to the substrate and the suspensor lines (Figs 1 and 2). Based on our field observations on the web-building behavior, these rail lines are produced without the intervention of the hind legs and, based on the small diameter, appear at first glance to lack cribellar fibrils compared to the ladder threads (Figs 1 and 2). However, the cribellate character of the rail lines is suggested by the blue appearance of the nanofibers using indirect lighting (Fig. 1B). Moreover, scanning electron microscopy shows that the rail lines are cribellate threads consisting of axial fibers and mostly tensed reserve warps, embedded in uncombed nanofibers (Fig. 2).

**Figure 3:**
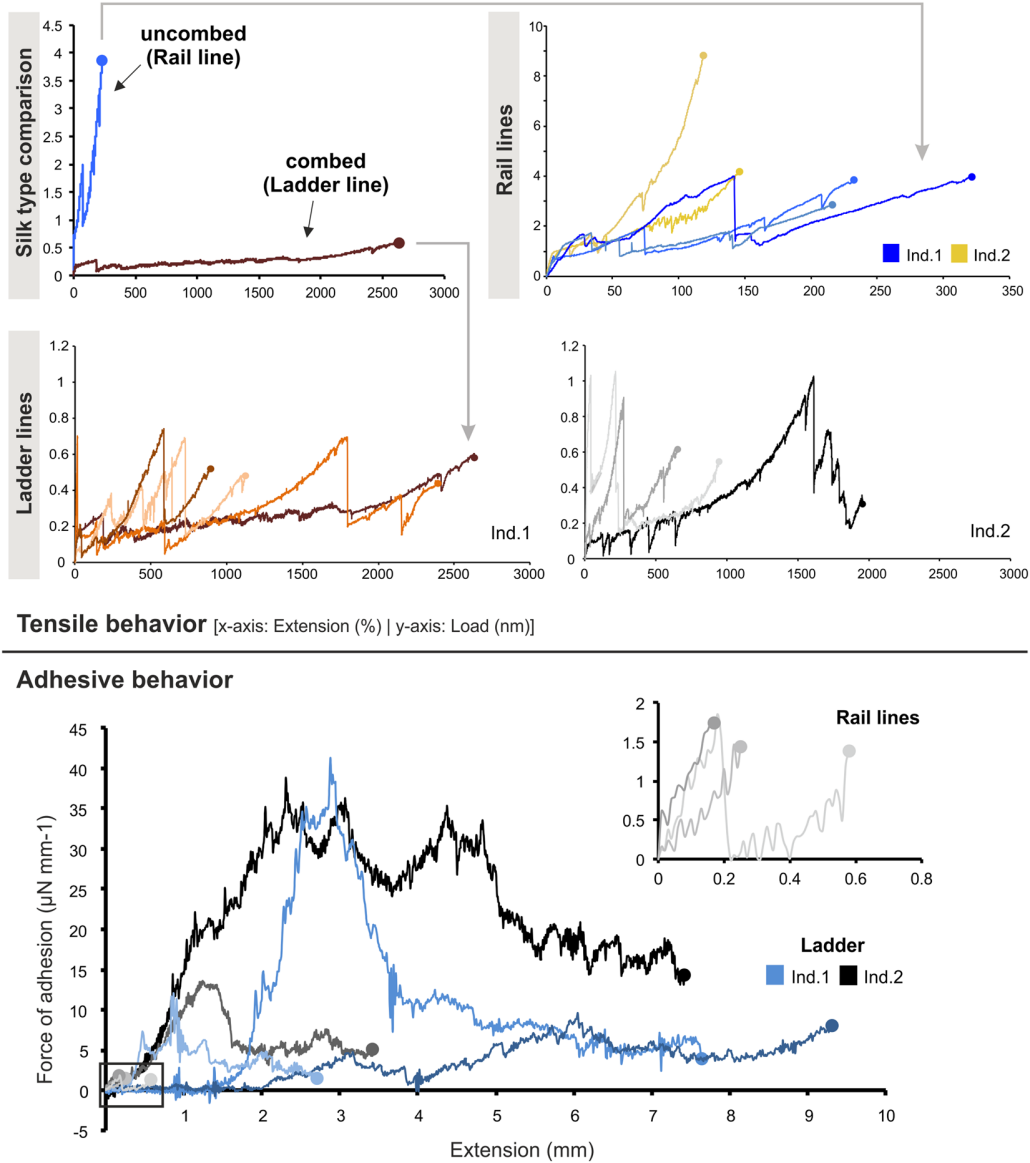
Tensile (top) and adhesive (bottom) behavior of silk from capture webs of *P*. *otwayensis*. The comparative graph shows the drastic difference in the tensile behavior of rail lines and ladder threads (grey arrows indicate to the corresponding measure). Color tones indicate replicate samples of a silk type from the same individual. Dots at the end of curves indicate fiber breaking or detaching from a glass substrate. Inset provide a higher resolution of low adhesion samples (bottom). Note the differences in the scaling of the axes for tensile behaviors.

Combed cribellate threads were very extensible, stretching well over 1.400% of their original length (Fig. 3; Table S1) while uncombed cribellate rail lines stretched only to about 200% (P < 0.001, Tables S2 and S3). However, rail lines were nearly 10x stronger (4.67 ± 0.69 vs. 0.56 ± 0.62 mN, P < 0.001, Table S3). Combed silk generated an order of magnitude more adhesive force (P = 0.04, Tables S4 and S5) and deformed more (5.47 ± 1.29 vs. 0.29 ± 1.39 mm, P < 0.001, Table S5) during pull-off to generate two orders of magnitude more work of adhesion than uncombed silk (76.03 ± 147.10 vs. 0.44 ± 169.22 nJ, P < 0.001, Table S5).

## Discussion

Cribellate silk is produced by an interplay of spinnerets, cribellum and the calamistrum on the hind legs, which combs the nanofibers extruded from the cribellum. Our data provide the first report of a spider spinning cribellate silk without the stereotypic combing behavior of the hind legs and show that cribellate silk can exhibit surprisingly diverse variation in performance controlled by spider spinning behavior. We suggest that uncombed cribellate silk in the web of *P*. *otwayensis* represents a new functional type of silk thread due to the clear differences in mechanical and adhesive properties compared to combed cribellate threads.

Tensile tests showed the composite nature of cribellate silk^6,19^. Based on the thread structure and load-extension behavior we hypothesize that threads were initially strong and stiff until the axial fibers broke then the threads continued to extend with erratic bumps and drops in stiffness as the coils in the reserve warp unwound until these fibers were completely tensed and threads again stiffened until reserve warp failure (Fig. 3). The difference in performance of combed and uncombed threads could relate to the degree to which these fibers deformed asynchronously. We hypothesize that in combed silk the axial fibers break before the excess slack is removed from the reserve warp and cribellate fibrils while the high alignment of fibers in uncombed threads result in simultaneous tensioning and therefore much stronger and stiffer, but less extensible performance.

The extensibility of *P*. *otwayensis* cribellate capture threads outperforms the highest performing spider capture silks (Table S1). But, this extensibility is largely achieved through the structure of the thread (e.g., the gradual uncoiling of the reserve warp and cribellate nanofibers)^6^ rather than through the extreme extensibility of the silk proteins seen in viscid capture silk^20^. Moreover, the uncombed structural cribellate thread also extend more than most other described structural threads in webs, such as typical ampullate draglines that only extend ~15–45% of their length (Table S2). The functional implications of such extensible structural threads remain to be tested.

Adhesive force generated by *Progradungula* capture threads is comparable to uloborid spiders (12–38 μN per mm, Table S4) and greater than other non-entelegyne species, such as *Hypochilus pococki* (6–7.5 μN per mm)^21–23^, although the magnitudes of these values may change on natural insect surfaces where cribellate nanofibers interact with epicuticular waxes to generate additional capillary forces^14^. The over ten-fold higher adhesion of combed silk in the ladder lines compared to uncombed silk in the rail lines is likely due to the increased surface area of cribellar puffs^16,23^. Combing therefore mediates a tradeoff in silk performance that allows the same fibers to act either as a relatively stiff structural element in webs (rail lines) or as very extensible adhesive elements (ladder lines).

Because the cribellum and calamistrum are always found together in spiders^24^, it is hard to imagine intermediate evolutionary morphologies that lead to this functionally correlated system. Our finding of cribellate silk produced without the intervention of the calamistrum relaxes the idea of an obligate functional correlation between both structures. At the same time, certain spiders are known to use special combs of setae in the hind tarsi to draw viscid silk from the anterior lateral spinnerets and wrap their prey^25,26^, thus it is conceivable that either precursor of the cribellum or the calamistrum may have appeared first in evolution.

In summary, *P*. *otwayensis* achieves a functional differentiation of silk through behavioural manipulation of the same silk type (combing vs. not combing), instead of using changes in silk protein expression typically seen in other species. The implications of replacing major ampullate structural threads with uncombed cribellate silk remain to be tested but the rail lines are much more extensible than ampullate silk, which likely has important implications for how these webs deform during prey capture and hence for the foraging ecology of these spiders. Prey capture behavior in *P*. *otwayensis*^27^ is similar to net-casting spiders (Deinopidae) where the web is highly elastic and actively used to ensnare prey^28^. This hunting strategy utilizes structural threads with high extensibility. Here we show that *P*. *otwayensis* has converged on highly extensible structural threads but by modifying the spinning behavior of capture silk rather than employing physiologically distinct silk types.

## Material and Methods

*Progradungula otwayensis* silk was collected as described in^29^ at Great Otway National Park, Victoria, Australia. Silk images were obtained using the BK PLUS Lab system (Dun Inc., USA) with a customized microscope lens (Ocellus) and 10x Mitutoyo objective mounted on a Canon 7D Mark II camera. Image stacks were processed using Zerene Stacker. Scanning electron microscope images were obtained with a field emission Zeiss Supra 40 in high vacuum, after sputter coating with AuPd.

Tensile mechanics of seven combed and five uncombed threads from two individuals were determined using a Nano Bionix® tensile tester (MTS) to generate load-extension data as previously described^6^. The low sample size is due to the rarity of this Australian endemic species, but still clearly demonstrates dramatic differences in silk properties. Samples were stretched at 1.5% extension s^−1^ at ambient temperature (~25 C) and humidity (~30% RH). Extensibility was calculated as percent change in length and represents structural changes as well as material properties due to the multiple components of the silk composite coming under tension at different times.

Adhesive properties of three combed and two uncombed threads from two individuals were determined using a Nano Bionix (MTS) similar to previous studies^17,30^. Cribellate threads were mounted perpendicularly above a 2 mm wide glass stage on the force plate. Fibers were pre-loaded to 50 μN for 10 seconds then pulled off at 0.1 mm s^−1^.

We analyzed our data using two general linear mixed models in the R package ‘lmerTest’. Adhesion data (force and extension at detachment) was log_10_ transformed data and analyzed with one random factors and a fixed effect. Tensile data (load and extension at breaking) was not transformed and analyzed with one random factor and fixed effect.

## Supplementary information

Supplemental Tables

## Data Availability

Additional tables supporting this article have been uploaded as part of the electronic Supplementary Material.
