# Supplementary material for: Functional trade-offs in cribellate silk mediated by spinning behavior
Source: Sci Rep. 2019 Jun 24;9:9092. doi: 10.1038/s41598-019-45552-x (PMC6591232; doi:10.1038/s41598-019-45552-x)
Supplement: Supplementary file 1 — Supplemental Tables [file 41598_2019_45552_MOESM1_ESM.pdf]

Supplementary Information:

Functional trade-offs in cribellate silk mediated by spinning behavior

Peter Michalik[1*], Dakota Piorkowski[2], Todd A. Blackledge[3] and Martín J. Ramírez[4]

[1]Zoological Institute and Museum, University of Greifswald, Germany

[2]Department of Life Sciences, Tunghai University, Taichung, Taiwan

[3]Department of Biology and Integrated Bioscience Program, The University of Akron, Akron OH, USA

[4]Division of Arachnology, Museo Argentino de Ciencias Naturales - CONICET, Buenos Aires, Argentina

***Authors for correspondence:**

Peter Michalik

Email: michalik@uni-greifswald.de

Table S1 - Extensibility of spider capture silks in comparison to *P. otwayensis*

Silk type	*Spider*	Sample size (N spiders, n silks)	Extensibility (%)	Reference
Cribellate	*P. otwayensis*	2, 7	1442.88 ± 199.2	This study
	Octonoba sinensis	18, 3	753	Opell & Bond 2000
	Uloborus glomosus	28, 3	604	
	Waitkera waitakerensis	24, 3	299	
	Deinopis spinosa	5, 27	511	Blackledge &Hayashi 2006a
	Hyptiotes cavatus	3, 22	118	
	Hyptiotes gertschi	2, 24	88	
	Uloborus diversus	11, 81	159	
	Deinopis spinosa	5, 27	105	Swanson et al. 2007
	Hyptiotes cavatus	3, 22	54	
	Hyptiotes gertschi	2, 24	49	
	Uloborus diversus	15, 103	52	
Viscid	*Araneus marmoreus*	10, 30	1039	Opell & Bond 2000
	Neoscona hentzii	19, 57	997	
	Cyclosa conica	20, 60	749	
	Argiope trifasciata	21, 63	1100	
	Microathena gracilis	15, 45	586	
	Argiope argentata	6, 50	410	Swanson et al. 2007
	Argiope aurantia	4, 41	385	
	Araneus diadematus	4, 33	362	
	Cyclosa turbinata	7, 44	437	
	Leucauge venusta	8, 66	86	
	Metepeira grandiosa	7, 50	274	
	Micrathena sagittata	5, 37	617	
	Neoscona oaxacensis	5, 49	301	
	Nephila clavipes	8, 45	517	
	Araneus marmoreus	3, -	326	Agnarsson & Blackledge 2009
	Araneus trifolium	7, -	458	
	Argiope aurantia	16, -	390	
	Argiope trifasciata	15, -	326	
	Cyclosa conica	2, -	139	
	Eustala sp.	3, -	175	
	Gasteracantha cancriformis	11, -	161	
	Larinioides cornutus	26, -	249	
	Lecauge venusta	12, -	144	
	Mangora maculata	9, -	139	
	Metepeira labyrinthea	9, -	180	
	Micrathena gracilis	16, -	146	
	Neoscona arabesca	35, -	210	
	Neoscona crucifera	5, -	339	
	Nephila clavipes	12, -	357	
	Tetragnatha sp.	4, -	192	
	Verrucosa arenata	4, -	219	
	Araneus diadematus	4, 16	290	Sensenig et al. 2010
	Araneus marmoreus	5, 20	326	
	Araneus trifolium	7, 28	458	
	Argiope aurantia	17, 68	381	
	Argiope trifasciata	9, 36	322	
	Caerostris darwini	7, 28	175	
	Cyclosa conica	5, 20	183	
	Eustala sp.	3, 12	175	
	Gasteracantha cancriformis	11, 44	161	
	Larinioides cornutus	26, 64	256	
	Leucauge venusta	12, 48	144	
	Mangora gibberosa	9, 36	139	
	Metepeira labyrinthea	9, 36	180	
	Micrathena gracilis	15, 60	153	
	Neoscona arabesca	15, 60	222	
	Neoscona crucifera	5, 20	339	
	Neoscona domiciliorum	4, 16	210	
	Nephila clavipes	13, 52	357	
	Nuctenea umbratica	7, 28	229	
	Tetragnatha versicolor	4, 16	148	
	Verrucosa arenata	8, 32	229	
	Zygiella x-notata	8, 32	192	

Values presented as mean (this study mean ± SE)

Table S2 - Extensibility of structural silks used by web building spiders in comparison to *P. otwayensis*

Silk type	Silk collected	*Spider*	Sample size (N spiders, n silks)	Extension (%)	Reference
Frame composite	Frame thread	*P. otwayensis*	2, 5	206.80 ± 235.10	This study
Dragline*	Orb web frame	*Araneus serratus*	-, 6	24	Denny 1976
	Orb web radial	*Araneus diadematus*	-, 15	40	Köhler & Vollrath 1995
	Web scaffolding	*Latrodectus hesperus*	9, 30	22	Moore & Tran 1999
	Orb mooring line	*Argiope trifasciata*	1, 28	30	Pérez-Rigueiro et al. 2001
	Gumfoot line	*Latrodectus hesperus*	7, 47	49	Blackledge et al. 2005
	Supporting thread		7, 54	42	
	Supporting thread	*Latrodectus hesperus*	9, 70	35	Swanson et al. 2006
	Lowering thread	*Hypochilus pococki*	10, 91	19	
	Lowering thread	*Holocnemus pluchei*	11, 105	16	
	Lowering thread	*Leucauge venusta*	6, 61	26	
	Lowering thread	*Metepeira grandiosa*	10, 88	27	
	Walking thread	*Kukulcania hibernalis*	12, 102	25	
	Walking thread	*Plectreurys tristis*	11,108	27	
	Walking thread	*Agelenopsis aperta*	10, 88	20	
	Walking thread	*Metaltella simoni*	6, 54	32	
	Orb web radial	*Araneus diadematus*	4, 16	39	Sensenig et al. 2010
	Orb web radial	*Araneus marmoreus*	5, 20	38	
	Orb web radial	*Araneus trifolium*	7, 28	36	
	Orb web radial	*Argiope aurantia*	17, 68	34	
	Orb web radial	*Argiope trifasciata*	9, 36	34	
	Orb web radial	*Caerostris darwini*	7, 28	39	
	Orb web radial	*Cyclosa conica*	5, 20	36	
	Orb web radial	*Eustala* sp.	3, 12	26	
	Orb web radial	*Gasteracantha cancriformis*	11, 44	49	
	Orb web radial	*Larinioides cornutus*	26, 64	31	
	Orb web radial	*Leucauge venusta*	12, 48	32	
	Orb web radial	*Mangora gibberosa*	9, 36	23	
	Orb web radial	*Metepeira labyrinthea*	9, 36	27	
	Orb web radial	*Micrathena gracilis*	15, 60	48	
	Orb web radial	*Neoscona arabesca*	15, 60	28	
	Orb web radial	*Neoscona crucifera*	5, 20	35	
	Orb web radial	*Neoscona domiciliorum*	4, 16	36	
	Orb web radial	*Nephila clavipes*	13, 52	32	
	Orb web radial	*Nuctenea umbratica*	7, 28	35	
	Orb web radial	*Tetragnatha versicolor*	4, 16	30	
	Orb web radial	*Verrucosa arenata*	8, 32	36	
	Orb web radial	*Zygiella x-notata*	8, 32	32	
	Orb web radial	*Caerostris darwini*	-, 30	39	Agnarsson et al. 2010
Major ampullate	Forcibly pulled			68	
	Forcibly pulled	*Uloborus diversus*	7, 61	26	Swanson et al. 2006
	Forcibly pulled	*Deinopis spinosa*	3, 24	21	
	Forcibly pulled	*Nephila clavipes*	17, 66	19	
	Forcibly pulled	*Araneus gemmoides*	3, 23	25	
	Forcibly pulled	*Mastophora hutchinsoni*	3, 21	31	
	Forcibly pulled	*Gasteracantha cancriformis*	3, 38	35	
	Forcibly pulled	*Argiope argentata*	8, 59	20	
Minor ampullate	Forcibly pulled	*Argiope trifasciata*	1, 8	55	Hayashi et al. 2004
	Forcibly pulled	*Argiope argentata*	8, 51	39	Blackledge & Hayashi 2006b
	Forcibly pulled	*Nephila inaurata*	2, 3	58	Guinea et al. 2012
	Forcibly pulled	*Argiope trifasciata*	2, 3	57	

Values presented as means (this study mean ± SE) (*) Dragline may contain multiple threads and is composed of major ampullate silk that may also contain minor ampullate silk

Table S3 - Results of general linear mixed model testing tensile properties of Progradungula silk

Property	Test	Estimate	SE	df	t-value	P
Load	Combed	0.5571	0.6231	1.6673	0.894	0.481371
	Uncombed - Combed	4.1152	0.7304	9.0066	5.634	0.000319
Extension	Combed	1442.89	199.19	10.07	7.244	<0.001
	Uncombed - Combed	-1236.09	308.59	10.07	-4.006	<0.001

Table S4 - Peak adhesive force of detachment (mean ± SE) of cribellate silk from various spiders

Spider	Silk function	Relative humidity (%)	Force (μN mm^{-1})	Reference
P. otwayensis	Capture	30	19.05 ± 1.29	This study
P. otwayensis	Structure (RL)	30	1.65 ± 1.38	
Hypochilus pococki	Capture	<2	6	Hawthorn & Opell 2002
H. pococki	Capture	>99	7	
Uloborus glomosus	Capture	<2	12	
U. glomosus	Capture	46	24	
U. glomosus	Capture	>99	30	
Hyptiotes cavatus1	Capture	<2	8	
H. cavatus1	Capture	46	22	
H. cavatus2	Capture	<2	27	
H. cavatus2	Capture	>99	38	
H. pococki	Capture	2	7.5	Hawthorn & Opell 2003
H. cavatus	Capture	2	25	
H. cavatus	Capture	99	37.5	

Force values are normalized by width of contact surface. Force values from references were approximated from figures. Values from Hawthorn & Opell (2003) were secondarily normalized for this table using reference contact length. (1,2) indicates samples collected from separate populations
RL = rail lines

Table S5 - Results of general linear mixed model testing log10-transformed data of adhesive properties of Progradungula silk

Property	Test	Estimate	SE	df	t-value	P
Force	Combed	1.28	0.11	6.00	11.61	<0.001
	Uncombed - Combed	-1.06	0.18	6.00	-5.86	0.001
Extension	Combed	0.74	0.11	6.00	6.60	<0.001
	Uncombed - Combed	-1.27	0.18	6.00	-6.98	<0.001
Work	Combed	-1.12	0.18	6.00	-6.27	<0.001
	Uncombed - Combed	-2.24	0.29	6.00	-7.68	<0.001

References

Agnarsson I, Blackledge TA (2009) Can a spider web be too sticky? Tensile mechanics constrains the evolution of capture spiral stickiness in orb-weaving spiders. *J Zool* **278**:134-140.

Agnarsson I, Kuntner M, Blackledge TA (2010) Bioprospecting Finds the Toughest Biological Material: Extraordinary Silk from a Giant Riverine Orb Spider. *Plos One* **5**.

Blackledge TA, Hayashi CY (2006a) Silken toolkits: biomechanics of silk fibers spun by the orb web spider Argiope argentata (Fabricius 1775). *The Journal of experimental biology* **209**:2452-2461.

Blackledge TA, Hayashi CY (2006b) Unraveling the mechanical properties of composite silk threads spun by cribellate orb-weaving spiders. *The Journal of experimental biology* **209**:3131-3140.

Blackledge TA, Swindeman JE, Hayashi CY (2005) Quasistatic and continuous dynamic characterization of the mechanical properties of silk from the cobweb of the black widow spider Latrodectus hesperus. *The Journal of experimental biology* **208**:1937-1949.

Guinea GV, Elices M, Plaza GR, Perea GB, Daza R, Riekel C, Agullo-Rueda F, Hayashi C, Zhao Y, Perez-Rigueiro J (2012) Minor Ampullate Silks from *Nephila* and *Argiope* Spiders: Tensile Properties and Microstructural Characterization. *Biomacromolecules* **13**:2087-2098.

Hawthorn AC, Opell BD (2002) Evolution of adhesive mechanisms in cribellar spider prey capture thread: evidence for van der Waals and hgroscopic forces. *Biol J Linn Soc* **77**:1-8.

Hawthorn AC, Opell BD (2003) van der Waals and hygroscopic forces of adhesion generated by spider capture threads. *Journal of Experimental Biology* **206**:3905-3911.

Hayashi CY, Blackledge TA, Lewis RV (2004) Molecular and mechanical characterization of aciniform silk: Uniformity of iterated sequence modules in a novel member of the spider silk fibroin gene family. *Mol Biol Evol* **21**:1950-1959.

Köhler T, Vollrath F (1995) Thread biomechanics in the two orb - weaving spiders *Araneus diadematus* (Araneae, Araneidae) and *Uloborus walckenaerius* (Araneae, Uloboridae). *Journal of Experimental Zoology* **271**:1-17.

Moore AMF, Tran K (1999) Material properties of cobweb silk from the black widow spider Latrodectus hesperus. *Int J Biol Macromol* **24**:277-282.

Opell BD, Bond JE (2000) Capture thread extensibility of orb-weaving spiders: testing punctuated and associative explanations of character evolution. *Biol J Linn Soc* **70**:107-120.

Sensenig A, Agnarsson I, Blackledge TA (2010) Behavioural and biomaterial coevolution in spider orb webs. *J Evol Biol* **23**:1839-1856.

Swanson BO, Blackledge TA, Hayashi CY (2007) Spider capture silk: performance implications of variation in an exceptional biomaterial. *J Exp Zool A Ecol Genet Physiol* **307**:654-666.
